# Insectivorous bat reproduction and human cave visitation in Cambodia: A perfect conservation storm?

**DOI:** 10.1371/journal.pone.0196554

**Published:** 2018-04-30

**Authors:** Thona Lim, Julien Cappelle, Thavry Hoem, Neil Furey

**Affiliations:** 1 Centre for Biodiversity Conservation, Royal University of Phnom Penh, Phnom Penh, Cambodia; 2 Epidemiology and Public Health Unit, Institut Pasteur du Cambodge, Phnom Penh, Cambodia; 3 UPR AGIRs, CIRAD-ES, Montpellier, France; 4 Fauna & Flora International, Phnom Penh, Cambodia; 5 Harrison Institute, Kent, United Kingdom; University of Western Ontario, CANADA

## Abstract

Cave roosting bats represent an important component of Southeast Asian bat diversity and are vulnerable to human disturbance during critical reproductive periods (pregnancy, lactation and weaning). Because dramatic growth of cave tourism in recent decades has raised concerns about impacts on cave bats in the region, we assessed the reproductive phenology of two insectivorous species (*Hipposideros larvatus* sensu lato and *Taphozous melanopogon*) at three caves in Cambodia for 23 months in 2014–2016 and evaluated human visitation to these sites between 2007 and 2014. Despite the differing foraging strategies employed by the two taxa, the temporal consistency observed in proportions of pregnant, lactating and juvenile bats indicates that their major birth peaks coincide with the time of greatest cave visitation annually, particularly for domestic visitors and namely during the Cambodian new year in April. They also reflect rainfall patterns and correspond with the reproductive phenology of insectivorous cave bats in Vietnam. These findings were predictable because 1) insect biomass and thus food availability for insectivorous bats are optimal for ensuring survival of young following this period, and 2) the Khmer new year is the most significant month for religious ceremonies and thus domestic cave visitation nationally, due to the abundance of Buddhist shrines and temples in Cambodian caves. While the impact of visitor disturbance on bat population recruitment cannot be empirically assessed due to lack of historical data, it is nonetheless likely to have been considerable and raises a conservation concern. Further, because growing evidence suggests that insectivorous cave bats exhibit reproductive synchrony across continental Southeast Asia where countless cave shrines are heavily frequented during April in Theravada Buddhist countries (e.g., Myanmar, Thailand, Cambodia and Laos), our results may have wider applicability in the region. We consequently advocate for increased emphasis on sustainable cave management practices in Cambodia and further investigations to determine whether our findings present a broader concern for cave bat conservation in Southeast Asia.

## Introduction

Bat populations take a relatively long time to recover from declines associated with humans due to their low annual reproductive rates [1]. Most cave-roosting taxa only bear one or two offspring per year [2] and because any disturbance of the relatively small and confined spaces that caves provide tends to affect the entire aggregation, this negatively affects their population recruitment [3]. Large numbers of cave bats are also often concentrated into only a few specific roost sites resulting in high potential for disturbance [4] which is exacerbated by their high roost fidelity [5]. As a result, cave tourism, which has burgeoned in East and Southeast Asia in recent decades, has dramatically increased threats to cave bats in these regions [6]. Development of caves for tourism typically involves the introduction of artificial lighting and physical alterations to the cave environment and alongside disturbance caused by their presence, cave visitors create significant fluctuations in temperature, relative humidity and carbon dioxide concentrations, all of which can lead to roost abandonment [6]. In a review of 225 subterranean sites in China for instance, it was found that recreational activities had pronounced detrimental effects on numbers of bat species and presence of species of special conservation concern [7]. Other studies have also raised concerns about the impact of cave tourism on Chinese and Vietnamese bats [8,9,10].

Because disturbance during pregnancy, lactation and weaning is widely recognized as highly detrimental to recruitment in cave bat populations [3,4,11,12], protection during these periods is central to their conservation. For instance, non-tacile disturbance by cave visits during reproduction can lead to: 1) outright death of young that lose their roost-hold and fall to the cave floor; 2) females abandoning the roost for less ideal sites where reproductive success may be reduced; and, 3) greater energy expenditure among females and less efficient energy transfer to young (translating into slower growth of young and increased foraging demands on females) [3,4]. As reproduction is energetically expensive [13], many bat species time the event so that lactation, the most costly stage [14], coincides with peak food availability. This peak may also occur during weaning for many species [15]. In the seasonal tropics, growing evidence suggests reproductive activity for many insectivorous bats is associated with rainfall, with lactation occurring during the peak rainy season [1]. In continental Southeast Asia, this has been confirmed for insectivorous cave bat species in Vietnam, Malaysia and Myanmar which exhibit restricted seasonal monoestry (one litter in a two month period per female/year: [16]) [10,17,18].

Knowledge of the Cambodian bat fauna has grown in recent years. Of the ≈74 bat species currently known to occur nationally, half (37) comprise species that are frequently found in caves and other subterranean sites [19]. Due to the solubility of calcium carbonate, caves occur in particularly high densities in limestone karst and the largest areas of karst in Cambodia are found in the western and southern portions of the country (Battambang and Banteay Meanchey provinces, and Kampot and Kep provinces, respectively). Small areas of limestone also outcrop along the Mekong River valley close to the Laos border in Stung Treng province, northern Cambodia [20]. Following field assessments of these three regions in 2014–2016 [19], half of all caves surveyed (45/98) were found to be affected to varying extents by development for tourism (23 caves, mostly Buddhist shrines visited by foreigners and Khmer) and solely domestic ritualistic purposes (22 caves). As the timing of insectivorous bat reproduction and temporal patterns of human cave visitation are undocumented in Cambodia, we predicted that the former would correspond with the primary reproductive period for insectivorous cave bats in Vietnam [March–July: 10] due to the similar climate and wet monsoon seasons of these countries. Our underlying hypothesis was that the reproductive phenology of insectivorous bats in Cambodia would directly reflect rainfall patterns due to their influence on insect biomass and hence reproductive success [1,18]. Due to the abundance of Buddhist shrines and temples in Cambodian caves [19], we also predicted that domestic cave visitation would be non-random in peaking during important ceremonial periods for the Buddhist movement. Our overall purpose was to determine if these patterns might collectively raise a concern for conservation of cave-roosting bats in Cambodia.

## Materials and methods

### Ethics statement

We captured and handled bats in the field in accordance with guidelines approved by the American Society of Mammalogists [21], in addition to the requirements of the statutory study permission provided by the national authority responsible for wildlife research, the Forestry Administration of the Cambodian Ministry of Agriculture, Forest and Fisheries. As an animal ethics committee did not exist at the time in Cambodia, all study aspects were overseen by the Forestry Administration who participated in the field investigations. Due to taxonomic uncertainties regarding the *H*. *larvatus* complex and morphological differences among bats at the study site (see below), collection of four voucher specimens was necessary to validate field identifications. Because anaesthetics currently favoured for euthanasia were not available in Cambodia at the time, diethyl ether was approved for this purpose. Small amounts of this agent were carefully used on three brief occasions in highly-ventilated outdoor settings in the field during which personal protective equipment was employed.

### Study site

**Fig 1:**
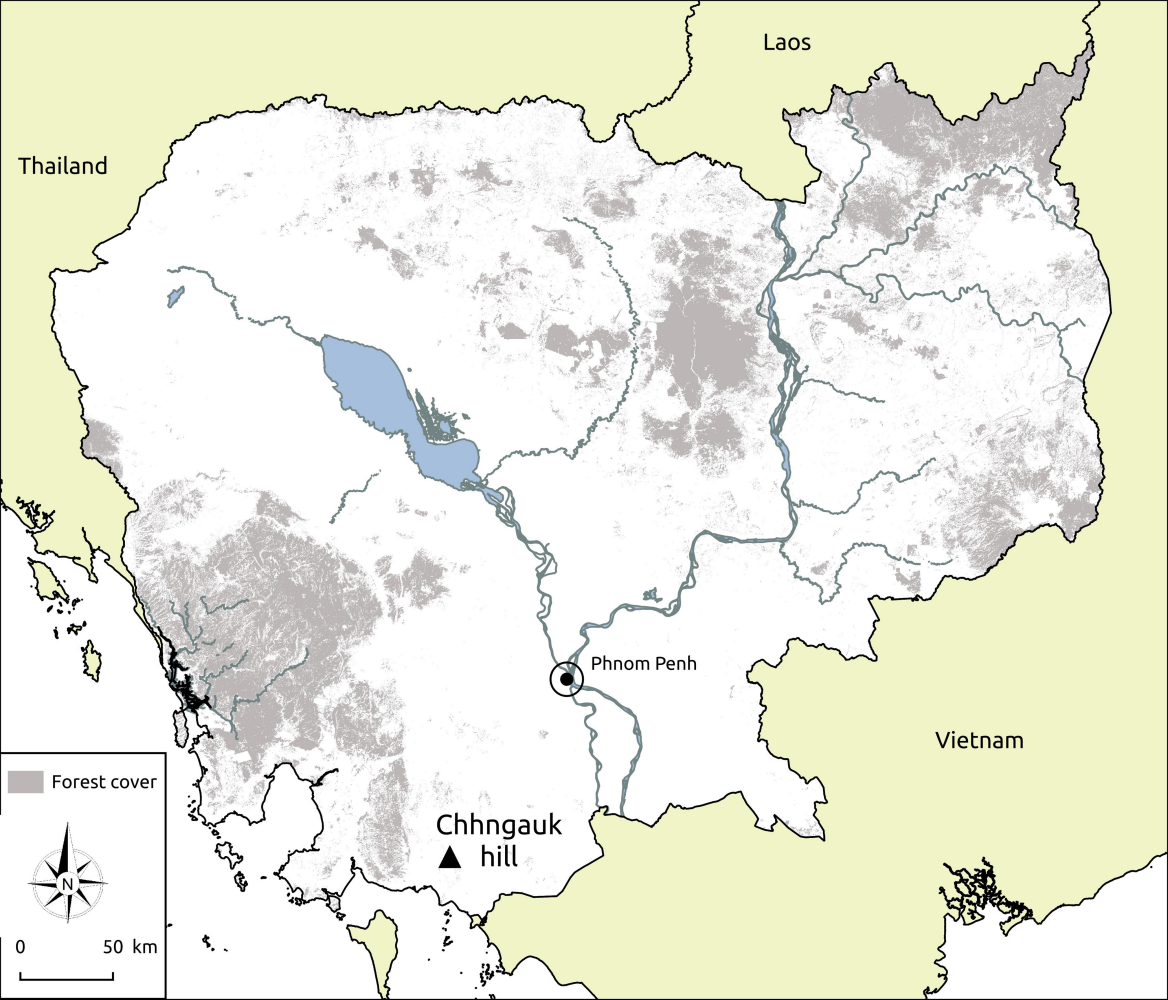
Location of Chhngauk hill in southern Cambodia.

Our field work was undertaken at Chhngauk hill (10.642601°N, 104.271412°E), a popular tourist site ca. 9 km north of the Cambodian coast (Fig 1) in Kampot province in 2014–2016. Chhngauk hill is a small (0.36 km^2^, elevation range: 20–130 m above sea level) and isolated karst outcrop surrounded by seasonal (rain fed) wet rice cultivation, with many local farmers also cultivating small quantities of durian, banana, mango, coconut, guava, and cashew. We chose the study site because its caves are representative of Cambodian caves in a wider context in harbouring nationally ubiquitous bat species and because their external environment is similar to that of karst hills throughout the country [19]. Local climate is monsoonal with an average annual rainfall of 1,920 mm and an average humidity of 79%. Temperature varies little throughout the year with mean monthly temperatures of 26–28°C and an annual average of 27°C. The wet season typically lasts from May to October and the dry season from November to April, with ≈80% of annual rainfall occurring during the former season.

Speleologists have registered and mapped six caves at Chhngauk hill [22,23] and we sampled three during the study: Trai Lak (or Ta Keav, 10.639462°N, 104.269647°E, length ≈50 m), Bat Khteas (10.644008°N, 104.269640°E, length 978 m), and Pota Am (or Pras Mea Kong Kea, 10.646497°N, 104.269245°E, length 356 m). Although all six caves include Buddhist shrines which are visited by people who reside outside the area, we did not sample the remaining three (Vihear Tuk-Bonn, Phum Tathy, and Vihear Tathor cave) because their size and/or structure precluded live-trapping of appreciable numbers of bats. Local authorities maintain visitor records for one cave (Vihear Tuk-Bonn) due to entrance fees which are charged for visitors to the late sixth-century Indian Shiva temple within the cave.

### Study species

We evaluated two bat species during the study: intermediate leaf-nosed bat *Hipposideros larvatus* (Horsfield, 1823) (Hipposideridae) and black-bearded tomb bat *Taphozous melanopogon* Temminck, 1841 (Emballonuridae). Both species are gregarious, form large diurnal roosts [24] and are among the most common bats in Cambodian caves [19].

Although *H*. *larvatus* represents a species complex [25], we adopt this name for the taxon at Chhngauk hill because the taxonomic status, diagnostic characters and correct names for each form await clarification [24]. As such, *H*. *larvatus* sensu lato (s.l.) occurs from northeastern South Asia, throughout much of southern China and continental Southeast Asia, into several islands within insular Southeast Asia [26]. The species is an aerial insectivore and roosts in caves, rock crevices, temples and old mines [24,27]. Its wing morphology indicates that it forages in partially cluttered to semi-open spaces such as clearings, streams, or other tunnels within forest or just above the forest canopy, although commuting bats are often caught in forest understory habitats [28].

*Taphozous melanopogon* is a medium-sized bat which occurs throughout much of South Asia, southern China and continental and insular Southeast Asia [29]. The species roosts in caves, rock crevices, temples and tree hollows [30,31]. Like many other tomb bats, *T*. *melanopogon* flies rapidly and high and forages for insects (including termite swarms [32]) on the wing in unobstructed airspaces over forests and other habitats, including highly disturbed areas [24]. This is reflected in its high aspect ratio wing and high wing loading [23] and as a consequence, the species is seldom captured at ground level away from its roost sites.

### Data collection

We obtained data on domestic and international visitation to Chhngauk hill (Vihear Tuk-Bonn cave) for January 2007 to May 2014 from monthly records maintained by the provincial Department of Tourism in Kampot (S1 Table). Data on international arrivals in Cambodia was also obtained for the same period from official tourism statistics [33,34] (S2 Table). One night of live-trapping was undertaken on consecutive nights at Trai Lak cave and Bat Khteas cave each month from February 2014 to January 2016 (except in May 2015) and at Pota Am cave from March 2015 to January 2016 (except May 2015).

We used mist nets (70 denier, 2 ply) of varying sizes (10x3m, 7x3m, 6x3m) to sample bat assemblages at the entrance of each cave or suitable locations inside. These were opened before sunset and attended constantly until ca. 1930 hrs (by which time most bats had exited the roost sites). Bats were measured and identified using field guides [24,27] and released unharmed near their capture site. Due to taxonomic uncertainties regarding species within the *H*. *larvatus* complex [24] and the absence of dark hairs on the chin of *Taphozous* bats at the study site (the character that *T*. *melanopogon* derives its vernacular name from), we retained two non-reproductively active adult bats of each species as voucher specimens to determine their identity. These were euthanized by placing each bat in a sealed plastic bag containing a small piece of cotton wool impregnated with diethyl ether and subsequently stored in ethanol. The specimens were prepared for comparative examination and revealed that the hipposiderids could not be assigned with confidence to any particular form within the *H*. *larvatus* complex [25], whereas the emballonurids matched descriptions of *T*. *melanopogon* in every other respect. All four specimens were deposited in the zoological reference collection at the Centre for Biodiversity Conservation (CBC), Royal University of Phnom Penh (accession numbers: *H*. *larvatus* s.l.—CBC 02360, CBC 02451; *T*. *melanopogon*—CBC 02352, CBC 02355).

### Reproductive diagnoses

We examined all bats to determine their sex, age and reproductive status [35,36]. Following similar studies [10], we classified young bats as non-volant pups if attached to their mother, whereas volant individuals lacking fully ossified and fused metacarpal-phalangeal epiphyses were classified as juveniles. The latter were recognised by examination of the epiphyses (aided by trans-illumination) as the cartilaginous epiphyseal plates indicative of juvenility in insectivorous bats are typically visible up until approximately two months of age [35]. Genitalia were examined to determine the status of non-juvenile males and these were classified as reproductively immature if they lacked enlarged testes and/or distended caudae epididymides, or mature if these were enlarged or distended [36].

We classified non-juvenile females as nulliparous or parous, and, reproductively inactive, pregnant and/or lactating. This was determined by examining the development and morphology of mammary glands and thoracic (axillary) nipples, and pubic nipples (in the case of *H*. *larvatus* s.l.). Pregnancy was assessed by abdominal palpation to determine the presence of foetuses. Lactation was confirmed through the extrusion of milk following gentle palpation of the mammary glands and nipples.

### Analysis

Data from the same month in separate years were combined for each species in analyses. Relationships between normally distributed data (international cave visitation and international arrivals in Cambodia) were investigated using Pearson’s product-moment correlation (*r*), whereas relationships between non-normally distributed data (rainfall and lactation) were investigated using Spearman’s rank-order correlation (*r*_s_). As domestic cave visitation data were not normally distributed, Kruskal–Wallis tests were employed for monthly comparisons, with post hoc testing using pairwise Mann–Whitney U tests.

## Results

We captured a total of 955 bats of the two target species (*H*. *larvatus* s.l.: 640 bats, *T*. *melanopogon*: 315 bats) during the study (S3 Table). Both species were captured every sampling month with a monthly mean of 28 bats (SD ±18.57) for *H*. *larvatus* s.l. (min–max: 4–65 bats) and 14 bats (SD ±8.97) for *T*. *melanopogon* (min–max: 1–37), although captures of both species were noticeably greater in the first study year compared to the second (*H*. *larvatus* s.l.: 388 vs. 252 bats, *T*. *melanopogon*: 208 vs. 107 bats). This was due to intensive hunting which was reported by local residents on several occasions early in 2015 (including at least one event when hundreds of bats were killed) and was confirmed by unequivocal evidence of hunting found in the caves.

### Reproductive phenology

**Fig 2:**
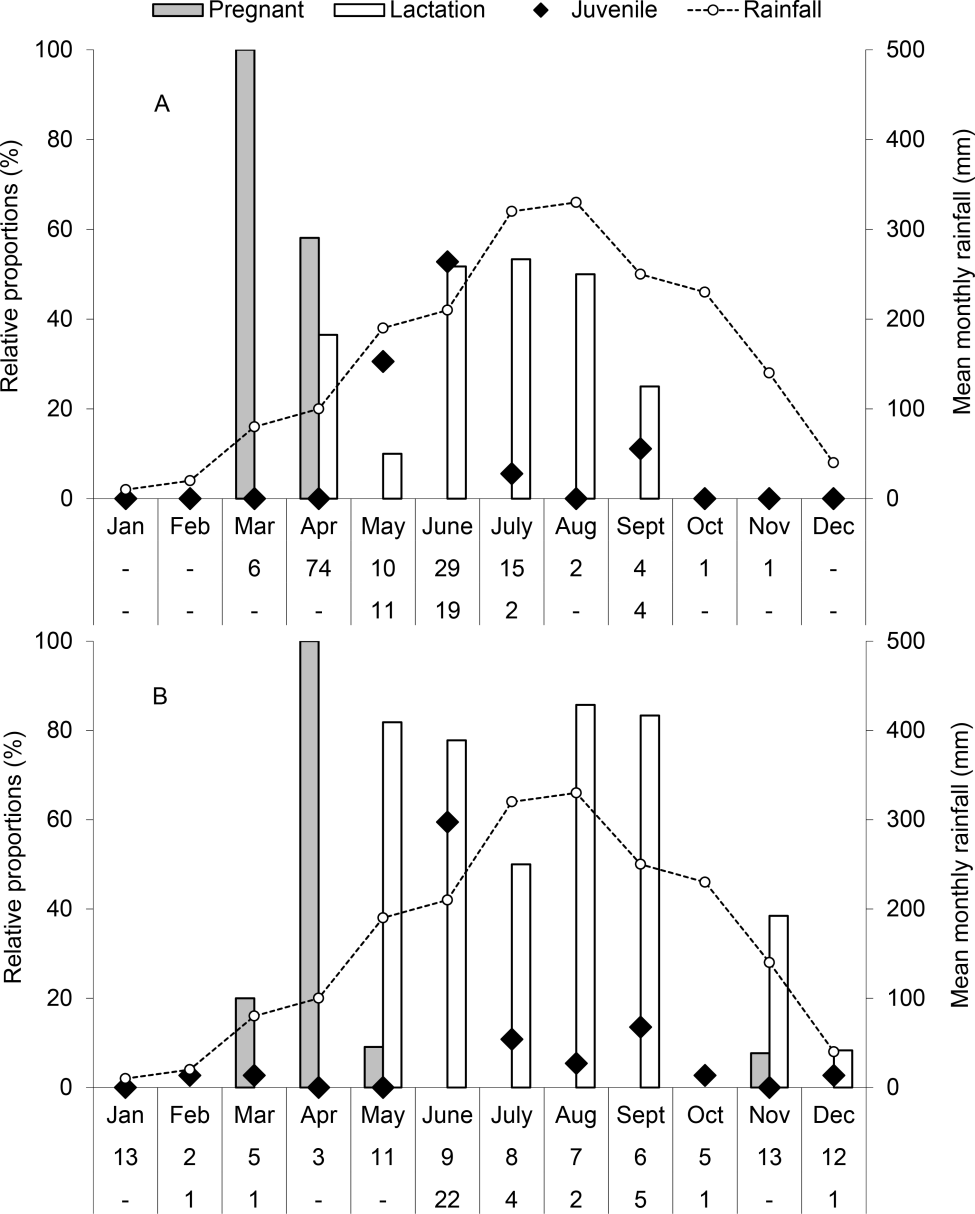
**Reproduction of a) *Hipposideros larvatus* s.l. and b) *Taphozous melanopogon* at Chhngauk hill from February 2014 to January 2016 in relation to monthly rainfall (44 year means) in southern Cambodia.** The first row of figures below each graph represents the total number of parous, pregnant and lactating females caught each month and the second row represents the same for juveniles. The ‘pregnant’ category is confined to non-lactating pregnant bats, while the ‘lactation’ category includes all lactating bats, whether pregnant or not. Relative proportions for juveniles were derived by dividing the respective monthly total by the study total × 100. The figures are based upon captures of a) 178 bats and b) 131 bats, and data from the same month in each study year are combined for each species.

Pregnancy in *H*. *larvatus* s.l. was confined to March–April and lactation to April–September (Fig 2A). Monthly percentages of lactating females were positively correlated with rainfall (*r*_s_ = 0.705, *p* = 0.01). Because 13 females of the species were observed carrying non-volant pups in April (seven in 2014, six in 2015) and most juveniles were recorded two months later in June of both years (53%, 19 of 36 overall), the majority of *H*. *larvatus* s.l. births appear to occur around April. As juveniles were recorded from May to July and also in September however, births evidently also occur after April and weaning is not complete until the later month each year. As such, the reproductive pattern of *H*. *larvatus* s.l. in southern Cambodia may be classified as extended seasonal monoestry (one litter per female/year within a 2–7 month period).

Pregnancy in *T*. *melanopogon* was observed in March–May and November, whereas lactation was observed in May–September and November–December (Fig 2B). Monthly percentages of lactating females were positively correlated with rainfall (*r*_s_ = 0.725, *p* = 0.008). Because eight females were observed carrying non-volant pups in May 2014 and most juveniles were recorded in June of both study years (59%, 22 of 37 overall), the majority of births also appear to occur around April. However, as pregnancy and lactation were also recorded in November and November–December respectively, a second cohort is evidently also borne in the last quarter of the year. Further, because low numbers of juveniles were observed in all but four months of the year (January, April–May, and November), the reproductive pattern of *T*. *melanopogon* in southern Cambodia may be classifiable as seasonal or continuous bimodal polyoestry (two litters per female/year in two distinct seasons of parturition), although it is also possible that different females breed at different times of the year.

### Human cave visitation

**Fig 3:**
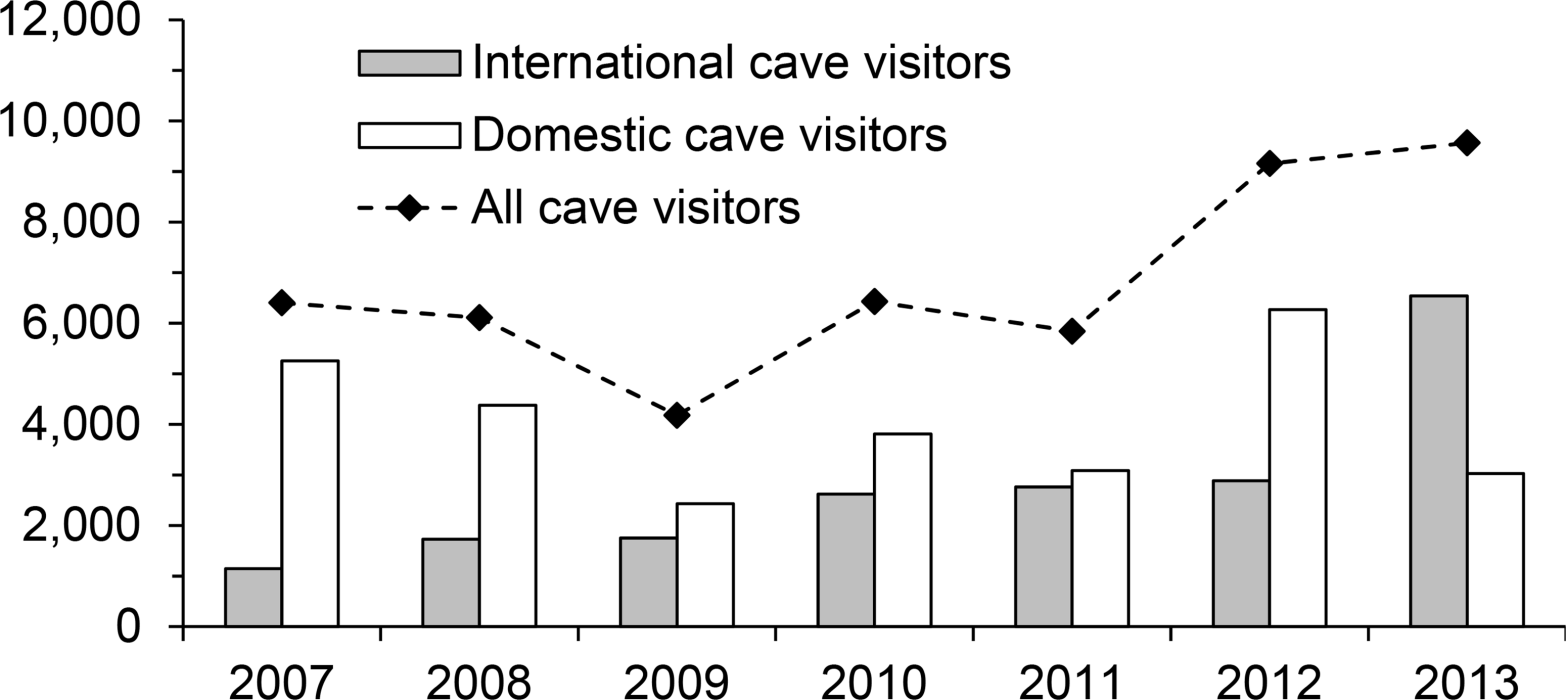
Annual visitation to Vihear-Tuk Bonn cave (Chhngauk hill, southern Cambodia) from January 2007 to December 2013.

**Fig 4:**
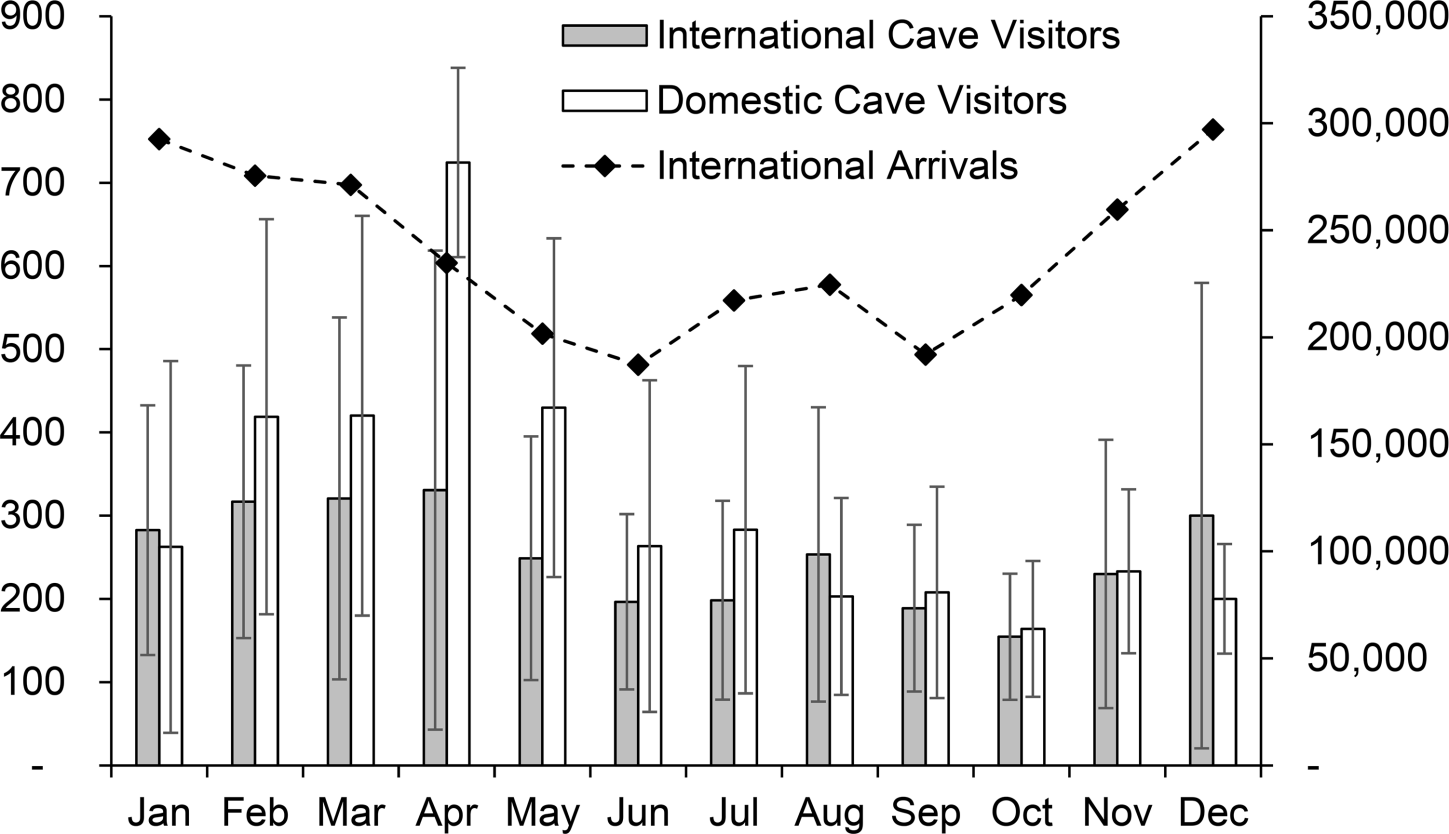
Mean monthly visitation to Vihear-Tuk Bonn cave (Chhngauk hill, southern Cambodia) and mean monthly international arrivals in Cambodia from January 2007 to May 2014. Vertical whiskers represent standard deviations.

Annual numbers of visitors to Chhngauk Hill (Vihear Tuk-Bonn cave) increased from 6,403 visitors in 2007 to 9,567 in 2013 (49%) (Fig 3). Annual figures for domestic (Khmer) visitors varied markedly during this period (ranging from 2,429 to 6,262 /year), whereas the same figures for international visitors consistently increased each year and almost six-fold overall from 1,147 in 2007 to 6,539 in 2013. From January 2007 to May 2014, Khmer accounted for 40–69% of mean monthly cave visitors. Khmer cave visitation was non-random with the greatest numbers visiting in February–May (monthly visitor means ±SD: 419 ±237–724 ±114) and these peaking significantly in April (the Khmer new year) compared to months outside of February–May (Mann–Whitney U, all values of *p* < 0.05) (Fig 4). Over the same period, mean monthly figures for international visitors were less variable, although somewhat higher in December–April (283 ±150–331 ±228) compared to other months (155 ±76–253 ±177). Monthly figures for international cave visitors were positively correlated with international arrivals in Cambodia over the same period (*r* = 0.687, *p* = 0.014).

## Discussion

Despite relatively small sample sizes and the different foraging environments used by *H*. *larvatus* s.l. and *T*. *melanopogon*, the temporal consistency in proportions of pregnant, lactating and juvenile bats observed during the study clearly supports our prediction that the reproductive phenology of these species reflects rainfall patterns and corresponds with the reproductive phenology of insectivorous cave bats in Vietnam. It is also evident that the major birth peaks for our study taxa coincide with the time of greatest cave visitation annually at Chhngauk hill, particularly for domestic visitors and specifically during the Khmer new year in April.

This is expected for two reasons. First, our finding that insectivorous cave bats in Cambodia bear young at the beginning of the wet season (May to October) and lactate for most of its duration is consistent with predictions [1] for the seasonal tropics. This accords with the findings of similar research in Vietnam [10] and is because insect abundance and thus food availability are optimal for ensuring reproductive success during this period [37,38,39,40,41]. We consequently anticipate that future research will reveal a similar pattern for other insectivorous cave bats in Cambodia and this is borne out by opportunistic capture data for multiple species throughout the country in 2009–2014 and more recently by intensive monthly sampling of the cave-roosting molossid *Chaerephon plicatus*. Further, because seasonal areas in continental Southeast Asia are largely dominated by the southwest monsoon which results in heavy rainfall from May to October, and subsequently by the northeast monsoon from November to April when rainfall is scant [42], we suspect that the same will also prove true for many insectivorous cave bats in the region. Growing evidence from a variety of published and unpublished research indicates that this is in fact the case [10,17,27,43,44] (S1 and S2 Texts) and despite less seasonality in rainfall, that insectivorous cave bat species also bear young at the same time in regions such as Peninsular Malaysia [18] and Indonesia [45].

Second, because Khmer new year in April is the most significant national holiday in Cambodia (a predominantly Buddhist country [46]) and coincides with or follows the dry season rice harvest [47], this is the most important month for religious ceremonies and thus domestic cave visitation, due to the abundance of shrines and temples in Cambodian caves [19,48]. The association between Buddhism and caves is fundamental [49] and dates back to the very beginning of the religion, when according to Buddhist tradition, the first council of 500 followers after the Masters death took place at the start of the Buddhist era in 543 BC in Saptaparna Guha cave, Nepal [50]. Temples and places of worship like those at Chhngauk hill consequently abound in and on limestone hills throughout continental Southeast Asia [51,52] and are heavily frequented for religious ceremonies and/or recreation during the new-year period. In predominantly Theravada Buddhist countries (Myanmar, Thailand, Cambodia & Laos), the new year is celebrated in April, whereas countries of more mixed religion such as Vietnam follow a lunisolar calendar whereby the most important period for ceremonies and domestic cave visitation occurs during the Tet festival in January–March [6,46,48] (S3 Text). In addition, our finding that numbers of international visitors to caves are typically higher in December–April is also predictable, being consistent with longstanding monthly patterns of international arrivals in Cambodia [33,34].

While the impact of visitor disturbance on population recruitment cannot be empirically assessed due to the absence of historical data for cave bat populations in Cambodia, it is nonetheless likely to have been considerable and raises a conservation concern. Uncontrolled human disturbance often leads to decreases in numbers of bats roosting in caves [53] and numerous studies have demonstrated the detrimental effects of cave tourism in particular [7,54,55,56,57,58]. Because many caves in Cambodia are affected to varying extents by development for tourism and domestic ritualistic purposes [19], we advocate for increased emphasis on sustainable cave management practices [59] nationally, particularly for tourist sites (which almost invariably comprise Buddhist shrines visited by foreigners and nationals [19]). This would be consistent with the significant growth of support for environmental issues within the Buddhist movement and other major world religions in recent years [60,61], and above all, site management should attempt to minimise disturbance to cave bat colonies during critical reproductive periods (pregnancy, lactation and weaning). For instance, this could be pursued through practices such as: prohibiting visitors to roost locations during maternity periods; ensuring visits by small groups, accompanied by a guide; timed visits, limiting disturbance; and avoiding or reducing lighting, noise, trash, and other anthropogenic disturbance as much as possible [62]. In addition, while the number of caves developed for religious worship and/or tourism in other countries could constitute a smaller proportion of the total number of caves present compared to Cambodia, the extent to which temporal patterns in insectivorous bat reproduction and human visitation present a concern for cave bat conservation more broadly in Southeast Asia also warrant investigation.

## Supporting information

S1 Table Domestic and international visitation to Chhngauk hill (Vihear Tuk-Bonn cave) from January 2007 to May 2014 inclusive. (XLSX)

S2 Table International arrivals in Cambodia from January 2007 to May 2014 inclusive. (XLSX)

S3 Table Sex, age and reproductive status of *Hipposideros larvatus* s.l. and *Taphozous melanopogon* captured at Chhngauk hill from February 2014 to January 2016 inclusive (except May 2015). (XLSX)

S1 Text Bounsavane Douangboubpha, personal communication, 8 March 2017. (PDF)

S2 Text Pipat Soisook, personal communication, 10 March 2017. (PDF)

S3 Text Vuong Tan Tu, personal communication, 10 March 2017. (PDF)

## References

[1] Racey PA, Entwistle AE. Life history and reproductive strategies of bats In: Crichton EG, Krutzsch PH, editors. Reproductive biology of bats. San Diego: Academic Press; 2000 pp. 363–468.

[2] Barclay RMR, Harder LD. Life histories of bats: Life in the slow lane In: Kunz TH, Fenton MB, editors. Bat ecology. Chicago: The University of Chicago Press; 2003 pp. 209–253.

[3] McCracken GF. Cave conservation: Special problems of bats. American National Speleological Society Bulletin. 1989;51: 47–51.

[4] Sheffield SR, Shaw JH, Heidt GA, McQenaghan LR. Guidelines for the protection of bat roosts. J Mammal. 1992;73: 707–710.

[5] Lewis SA. Roost fidelity of bats: A review. J Mammal. 1995;76: 481–496.

[6] Furey NM, Racey PA. Conservation ecology of cave bats In: Voigt CC, Kingston T, editors. Bats in the Anthropocene: Conservation of bats in a changing world. Switzerland: Springer; 2016 pp. 463–500.

[7] Luo J, Jiang T, Lu G, Wang L, Wang J, Feng J. Bat conservation in China: Should protection of subterranean habitats be a priority? Oryx. 2013;47: 526–531.

[8] Niu H, Wang N, Zhao L, Liu J. Distribution and underground habitats of cave-dwelling bats in China. Anim Conserv. 2007;10: 470–477.

[9] Zhang L, Zhu G, Jones G, Zhang S. Conservation of bats in China: Problems and recommendations. Oryx. 2009;43: 179–182.

[10] Furey NM, Mackie IJ, Racey PA. Reproductive phenology of bat assemblages in Vietnamese karst and its conservation implications. Acta Chiropt. 2011;13: 341–354.

[11] Jubertie C. Conservation of subterranean habitats and species In: Wilkens H, Culver DC, Humphreys WF, editors. Ecosystems of the world: Subterranean ecosystems. Amsterdam: Elsevier Science Publishers; 2000 pp. 691–700.

[12] Mitchell-Jones AJ, Bihari Z, Masing M, Rodrigues L. Protecting and managing underground sites for bats. Bonn: UNEP/EUROBATS Secretariat; 2007.

[13] Racey PA, Speakman JR. The energy costs of pregnancy and lactation in heterothermic bats. In: Loudon ASI, Racey PA, editors. Reproductive energetics in mammals. Symp Zool Soc Lond. 1987;57: 107–125.

[14] Kurta A, Bell GP, Nagy KA, Kunz TH. Energetics of pregnancy and lactation in free-ranging little brown bats (*Myotis lucifugus*). Physiol Zool. 1989;62: 804–818.

[15] Cumming GS, Bernard RTF. Rainfall, food abundance and timing of parturition in African bats. Oecologia. 1997;111: 309–317. doi: 10.1007/s004420050240 28308124 10.1007/s004420050240

[16] Happold DCD, Happold M. Reproductive strategies of bats in Africa. J Zool (Lond). 1990;222: 557–583.

[17] Khin Min Min Tun, Khin Mya Mya, Khin Muang Gyi. Reproduction and post-natal development of *Hipposideros pomona* Andersen, (1918) in Kyan Taing Aung cave of Sagaing hill range within Myanmar. J Trop Biol Conserv. 2015;12: 35–54.

[18] Nurul-Ain E, Rosli H, Kingston T. Resource availability and roosting ecology shape reproductive phenology of rainforest insectivorous bats. Biotropica. 2017; doi: 10.1111/btp.12464 29398713

[19] Furey N, Whitten T, Cappelle J, Racey PA. The conservation status of Cambodian cave bats In: Laumanns M, editor. International speleological project to Cambodia 2016 (provinces of Stoeng Treng, Kampong Speu, Banteay Meanchey and Battambang). Berlin: Berliner Höhlenkundliche Berichte; 2016 pp. 82–95.

[20] Laumanns M. International speleological project to Cambodia 2016 (Provinces of Stoeng Treng, Kampong Speu, Banteay Meanchey and Battambang) Berlin: Berliner Höhlenkundliche Berichte; 2016.

[21] Sikes RS, Gannon WL, and the Animal care and use committee of the American Society of Mammalogists. Guidelines of the American Society of Mammalogists for the use of wild animals in research. J Mammal. 2011;92: 235–253. 10.1093/jmammal/gyw078 PMC5909806 29692469

[22] Denneborg M, Laumanns M, Schnadwinkel M, Voigt S. German Speleological Campaign Cambodia 95/96 Berlin: Berliner Llohlenkundlicke Berichte; 2002.

[23] Lim T. Cave selection and reproductive phenology of insectivorous bats in southern Cambodian karst and its conservation implications. M.Sc Thesis, Royal University of Phnom Penh. 2016.

[24] Francis CM. A guide to the mammals of Southeast Asia New Jersey: Princeton University Press; 2008.

[25] Kruskop S. Dull and bright: cryptic diversity within the *Hipposideros larvatus* group in Indochina (Chiroptera: Hipposideridae). Lynx n.s. (Praha). 2015;46: 29–42.

[26] Bates P, Bumrungsri S, Suyanto A, Molur S, Srinivasulu C. Hipposideros larvatus. The IUCN red list of threatened species. 2008;1: 1 Available from: http://www.iucnredlist.org/details/10143/0.

[27] Kruskop S. Bats of Vietnam: Checklist and an identification manual 2nd ed Moscow: KMK Scientific Press; 2013.

[28] Furey NM, Racey PA. Can wing morphology inform conservation priorities for Southeast Asian cave bats? Biotropica. 2016; 48: 545–556.

[29] Csorba G, Bumrungsri S, Helgen K, Francis C, Bates P, Gumal M, et al Taphozous melanopogon. The IUCN red list of threatened species. 2008;1: 1 Available from: http://www.iucnredlist.org/details/21461/0.

[30] Bates PJJ, Harrison DL. Bats of the Indian subcontinent Kent: Harrison Zoological Museum; 1997.

[31] Kingston T, Lim BL, Zubaid A. Bats of Krau Wildlife Reserve Malaysia: Penerbit Universiti Kebangsaan Malaysia Bangi; 2006.

[32] Gould E. Opportunistic feeding by tropical bats. Biotropica. 1978;10: 75–76.

[33] Cambodian Ministry of Tourism. Tourism statistics report in 2011. Cambodian Ministry of Tourism. 2012; 1: 1–6. Available from http://www.tourismcambodia.org/mot/index.php?view=statistic_report.

[34] Cambodian Ministry of Tourism. Tourism statistics report, year 2015. Cambodian Ministry of Tourism. 2016; 1: 1–5. Available from http://www.tourismcambodia.org/mot/index.php?view=statistic_report.

[35] Anthony ELP. Age determination in bats In: Kunz TH, editor. Ecological and behavioral methods for the study of bats. Washington: Smithsonian Press; 1988 pp. 47–58.

[36] Racey PA. Reproductive assessment In: Kunz TH, Parsons S, editors. Behavioural and ecological methods for the study of bats. 2nd ed Baltimore: Johns Hopkins University Press; 2009 pp. 249–264.

[37] Frith CB, Frith DW. Seasonality of insect abundance in an upland Australian tropical rainforest. Aust J Ecol 1985;10: 237–248.

[38] McWilliam AN. Adaptive responses to seasonality in four species of microchiroptera in coastal Kenya. Ph.D Thesis, University of Aberdeen. 1982.

[39] McWilliam AN. The reproductive cycle of male tomb bats, *Taphozous hildegardeae* (Chiroptera: Emballonuridae), in a seasonal environment of the African tropics. J Zool (Lond). 1988;215: 433–442.

[40] Ades GWJ, Dudgeon D. Insect seasonality in Hong Kong: a monsoonal environment in the northern tropics. Memoirs of the Hong Kong Natural History Society. 1999;22: 81–97.

[41] Kai KH, Corlett RT. Seasonality of forest invertebrates in Hong Kong, South China. J Trop Ecol. 2002;18: 637–644.

[42] Goh KC. The climate of Southeast Asia In: Gupta A, editor. The physical geography of Southeast Asia. Oxford: Oxford University Press; 2005 pp. 80–93.

[43] Nu Nu Aye. Ecology and economic importance of Tadarida plicata (Buchannan, 1800), free-tailed bat in some areas of Myanmar. Ph.D Thesis, University of Yangon. 2006.

[44] Nguyen TS, O’Shea T, Gore JA, Csorba G, Vuong TT, Oshida T, et al Bats (Mammalia: Chiroptera) of the Southeast Truong Son Mountains, Quang Ngai province, Vietnam. J Threat Taxa. 2016;8: 8953–8969.

[45] Suyanto A, Struebig MJ. Bats of the Sangkulirang limestone karst formations, East Kalimantan—a priority region for Bornean bat conservation. Acta Chiropt. 2007;9: 67–95.

[46] CIA. The world factbook Washington: Central Intelligence Agency; 2016.

[47] GRiSP Rice almanac 4th ed Los Baños: International Rice Research Institute; 2013.

[48] Hays J. Theravada Buddhist holidays, calendar and religion ceremonies. Facts and details. 2013;1: 1 Available from: http://factsanddetails.com/asian/cat64/sub415/entry-2817.html.

[49] Sponsel L, Natadecha-Sponsel P. Illuminating darkness: the monk-cave-bat-ecosystem complex in Thailand In: Gottlieb RS, editor. This sacred earth: Religion, nature, environment. New York: Routledge; 2004 pp. 134–144.

[50] Sidisunthorn P, Gardner S, Smart D. Caves of northern Thailand Bangkok: River Books; 2006.

[51] Vermeulen JJ, Whitten AJ. Biodiversity and cultural property in the management of limestone resources Washington: The World Bank; 1999.

[52] Sponsel L. Sacred caves of the world: illuminating the darkness In: Brunn SD, editor. The changing world religion map: sacred places, identities, practices and politics. Netherlands: Springer; 2015 pp. 503–522.

[53] Tuttle MD. Threats to bats and educational challenges In: Adams RA, Pedersen SC, editors. Bat evolution, ecology, and conservation. New York: Springer; 2013 pp. 363–391.

[54] Stihler CW, Hall JS. Endangered bat populations in West Virginia caves gated or fenced to reduce human disturbance. Bat Res News 1993;34: 130.

[55] Mann SL, Steidl RJ, Dalton VM. Effects of cave tours on breeding *Myotis velifer*. J Wildl Manage. 2002;66: 618–624.

[56] Olson CR, Hobson DP, Pybus MJ. Changes in population size of bats at a hibernaculum in Alberta, Canada, in relation to cave disturbance and access restrictions. Northwest Nat 2011;92: 224–230.

[57] Cardiff SG, Ratrimomanarivo FH, Goodman SG. The effect of tourist visits on the behaviour of *Rousettus madagascariensis* (Chiroptera: Pteropodidae) in the caves of Ankarana, northern Madagascar. Acta Chiropt. 2012;14: 479–490.

[58] Furman A, Coraman E, Bilgin R. Bats and tourism: a response to Paksuz and Ozkan. Oryx 2012;46: 330.

[59] Hildreth-Werker V., Werker JC. Cave conservation and restoration Alabama: National Speleological Society; 2006.

[60] Sponsel L, Natadecha-Sponsel P. Buddhism and ecology In: Leeming DA, editor. Encyclopedia of psychology and religion. USA: Springer; 2016 pp. 214–219.

[61] Sponsel L, Natadecha-Sponsel P. Environment and nature: buddhism In: Selin H, editor. Encyclopedia of the history of science, technology, and medicine in non-western cultures. 2nd ed Netherlands: Springer; 2016 pp. 768–776.

[62] Medellin RA, Wiederholt R, López-Hoffman L. Conservation relevance of bat caves for biodiversity and ecosystem services. Biol Cons. 2017;211: 45–50.

