# Supplementary material for: Insectivorous bat reproduction and human cave visitation in Cambodia: A perfect conservation storm?
Source: PLoS One. 2018 Apr 30;13(4):e0196554. doi: 10.1371/journal.pone.0196554 (PMC5927413; doi:10.1371/journal.pone.0196554)
Supplement: S2 Text — (PDF) [file pone.0196554.s005.pdf]

Date: 10 March 2017

Re: Inclusion of a personal communication in the manuscript entitled “Insectivorous bat reproduction and human cave visitation in Cambodia: A perfect conservation storm?” by Thona Lim, Julien Cappelle, Thavry Hoem & Neil Furey.

To whom it may concern,

I am pleased to confirm that I fully support the inclusion of my personal communication with the above authors, specifically to the following effect in the text of their manuscript:-

“……… our finding that two insectivorous cave bat species in Cambodia bear young at the beginning of the wet season (May to October) and lactate for most of its duration is consistent with predictions [1] for the seasonal tropics. ……… Further, because seasonal areas in continental Southeast Asia are largely dominated by the southwest monsoon which results in heavy rainfall from May to October, and subsequently by the northeast monsoon from November to April when rainfall is scant [42], we suspect that the same will also prove true for many insectivorous cave bats in the region. Growing evidence from a variety of published and unpublished research indicates that this is in fact the case [10,17,27, 43,44] (Bounsavane Douangboubpha, National University of Laos pers. comm.; Pipat Soisook, Prince of Songkla University, Thailand pers. comm.) ………”

My support for these statements is based on over a decade of research experience of bats in Thailand, particularly insectivorous species which occasionally or frequently roost in caves.

Yours sincerely,

Pipat Soisook, PhD

Mammal Collection,
Princess Maha Chakri Sirindhorn Natural History Museum,
Prince of Songkla University
Hatyai, Songkhla
Thailand, 90112
Tel: +66 (0) 74 288065, +66 (0) 898761196
Email: pipat66@gmail.com, pipat.s@psu.ac.th
