# Supplementary material for: Insectivorous bat reproduction and human cave visitation in Cambodia: A perfect conservation storm?
Source: PLoS One. 2018 Apr 30;13(4):e0196554. doi: 10.1371/journal.pone.0196554 (PMC5927413; doi:10.1371/journal.pone.0196554)
Supplement: S3 Text — (PDF) [file pone.0196554.s006.pdf]

**Date:** 10 March 2017

**Re:** Inclusion of a personal communication in the manuscript entitled “Insectivorous bat reproduction and human cave visitation in Cambodia: A perfect conservation storm?” by Thona Lim, Julien Cappelle, Thavry Hoem & Neil Furey.

**To whom it may concern,**

I am pleased to confirm that I fully support the inclusion of my personal communication with the authors above, namely to the following effect in the text of their manuscript:-

“In predominantly Theravada Buddhist countries (Myanmar, Thailand, Cambodia & Laos), this [the new year] is celebrated in April, whereas countries of more mixed religion such as Vietnam follow a lunisolar calendar whereby the most important period for ceremonies and domestic cave visitation is during the Tet festival [6,46,48] (Vuong Tan Tu, Institute of Ecology and Biological Resources, Vietnam pers. comm.) in January–March.”

My agreement with this statement is based on more than a decade of research experience regarding cave-roosting bats and human-cave associations throughout Vietnam.

**Yours sincerely,**

Dr. Vuong Tan Tu
Institute of Ecology and Biological Resources,
Vietnam Academy of Sciences and Technology,
No. 18, Hoang Quoc Viet road, Cau Giay district, Hanoi, Vietnam.
Tel: 844 38 36 01 69 - Fax: 844 38 36 11 96. Email: vttu@iebr.ac.vn
